# Metabolomics of thyroid nodules and the future

**DOI:** 10.20945/2359-3997000000080

**Published:** 2018-10-01

**Affiliations:** 1 Universidade de São Paulo Universidade de São Paulo Faculdade de Medicina Departamento de Radiologia e Oncologia São Paulo SP Brasil Departamento de Radiologia e Oncologia da Faculdade de Medicina da Universidade de São Paulo (FMUSP), São Paulo, SP Brasil

In this issue of the *Archives of Endocrinology and Metabolism* (AE&M), the use of 3T Magnetic Resonance Spectroscopy (MRS) for the evaluation of patients with thyroid nodules (TNs) is addressed in a very interesting prospective study by Aghaghazvini and cols (1).

The new field in biological science, Metabolomics, makes use of analytical and standard recognition approaches and bioinformatics (2,3). The metabolome is the final downstream product of gene expression reflecting changes in transcriptome and proteome (4). Metabolic, histological and cytological changes occur during the development and progression of carcinoma (5), and it is paramount to understand its biochemistry to allow the development of powerful diagnostic tools and to identify new biomarkers (2). Several studies have demonstrated that the metabolomics makes it possible to characterize different types of tumors in other organs than thyroid gland (2).

Magnetic resonance spectroscopy (MRS) is a noninvasive method that analyzes tissue metabolism, allowing the measurement of the concentration of metabolites in tissues and organs and making it possible to characterize the metabolic changes associated with cancer (6).

As is known, standard magnetic resonance imaging (MRI) plays a limited role in the evaluation of thyroid nodules as well as does not assess its functional status (7,8). The main difference between the standard MRI and the MRS is that in the MRI the spatial distribution of the water proton signals is used to generate an anatomical image of the analyzed tissue and in the clinical MRS methods there is suppression of the water signal to provide chemical information about the metabolites in which the data are shown as line spectra (6,9), or maps of metabolic images if the MRS data are acquired using a method called chemical shift imaging. These spectra can be analyzed quantitatively by the presence, absence or alteration of metabolites and semi-quantitatively by the calculation of the amplitude or integrals of the metabolite or metabolite ratios relative to the control (6).

Although MRS is used to assess cancer in various regions of the body, the neck region may create technical difficulties such as shimming and subject motion for in vivo spectroscopy, and it is the only noninvasive technique capable of measuring chemicals/metabolites within the body (7,8). Thyroid cancer was the first field in which the molecular diagnosis was performed using ex-vivo MRS (10,11) in Fine- Needle Aspiration Biopsy (FNAB) specimen or tissue obtained at the time of surgery. Few in vivo studies were performed, most of them with 1.5 T MRS, even less with 3T MRS. There are technical difficulties in performing in vivo thyroid spectroscopy, such as tumor movement as a result of swallowing and breathing, shimming difficulties due to large differences in magnetic susceptibility between the neck and air in the trachea and contamination of spectra by adjacent fat (7).

In this study by Aghaghazvini and cols (1), the Choline (Chol) to creatine (Cr) ratio was assessed on each nodule, and the sensitivity (75%) is less than two main prior studies (King and cols. (12), 87%; Gupta and cols. (13), 100%) while the specificity is comparable to two studies. Further studies with larger number of patients are needed.

A review article by Minuto and cols. shows that there are other MRS studies in the literature demonstrating that benign and malignant thyroid lesions have a higher level of several amino acids (methionine, glycine, alanine, cysteine, glutamine, glutamate, isoleucine, leucine, lysine, phenylalanine, serine, tyrosine, valine), lactate and taurine, and a lower content of fatty acids than normal tissues. Papillary and follicular neoplasms have a higher concentration of taurine, lactate, phenylalanine and tyrosine, and lower concentrations of mi and scyllo-inositol, choline, phosphocholine and/or glycerophosphorus-line and unknown compounds in relation to follicular adenomas; increased levels of hypoxanthine and decreased levels of acetone in the thyroid lesions were also described, as well as new markers such as 3-hydroxybutyrate and -glucose and phosphocholine and formate. Four metabolites (creatine, scyllo-inositol, myo-inositol and uracil) were considerate candidates for selective biomarkers for thyroid cancer. The follicular thyroid adenomas have some metabolic characteristics of normal tissue and other features associated with thyroid cancer, which may denote an intermediate nature of these benign tumors, so that thyroid cancer may arise from preexisting follicular adenoma, or it may be a preneoplastic lesion (10).

Some metabolomic MRS studies have concluded that specific markers of benign and malignant tissues have been correlated with cell proliferation observed during tumor development (10).

The advancement in the MRI techniques as 3T and technology of software programs as MRS can improve the metabolomics of thyroid nodules in the future, perhaps even being a powerful complementary tool to other diagnostic methods.
